# Impact of Non-Accelerated Aging on the Properties of Parylene C

**DOI:** 10.3390/polym14235246

**Published:** 2022-12-01

**Authors:** Franz Selbmann, Christina Scherf, Jörn Langenickel, Frank Roscher, Maik Wiemer, Harald Kuhn, Yvonne Joseph

**Affiliations:** 1 Fraunhofer Institute for Electronic Nano Systems ENAS, Technologie-Campus 3, 09126 Chemnitz, Germany; 2 Institute for Electronic and Sensor Materials, TU Bergakademie Freiberg, Gustav-Zeuner-Straße 3, 09599 Freiberg, Germany; 3 Center for Microtechnologies, TU Chemnitz, Reichenhainer Straße 70, 09126 Chemnitz, Germany

**Keywords:** Parylene, aging, stability, barrier properties, FTIR, mechanical properties, nano-indentation, MEMS, encapsulation, packaging

## Abstract

The polymer Parylene combines a variety of excellent properties and, hence, is an object of intensive research for packaging applications, such as the direct encapsulation of medical implants. Moreover, in the past years, an increasing interest for establishing new applications for Parylene is observed. These include the usage of Parylene as a flexible substrate, a dielectric, or a material for MEMS, e.g., a bonding adhesive. The increasing importance of Parylene raises questions regarding the long-term reliability and aging of Parylene as well as the impact of the aging on the Parylene properties. Within this paper, we present the first investigations on non-accelerated Parylene C aging for a period of about five years. Doing so, free-standing Parylene membranes were fabricated to investigate the barrier properties, the chemical stability, as well as the optical properties of Parylene in dependence on different post-treatments to the polymer. These properties were found to be excellent and with only a minor age-related impact. Additionally, the mechanical properties, i.e., the Young’s modulus and the hardness, were investigated via nano-indentation over the same period of time. For both mechanical properties only, minor changes were observed. The results prove that Parylene C is a highly reliable polymer for applications that needs a high long-term stability.

## 1. Introduction

For the realization of ongoing trends, such as the “Internet of Things” (IoT), “Industry 4.0”, and “Smart Everything”, new concepts, technologies, and materials are required for the production of sensors and actuators in the form of microelectromechanical systems (MEMS). Particularly, the materials should enable miniaturization and an increasing performance of the device at the same time. Hence, materials beside the established silicon technology are in consideration.

Parylene (Poly-*p*-xylylene) is a family of thermoplastic polymers that is of increasing interest due to its combination of excellent properties. These include chemical inertness against all common acids, bases, and solvents, dielectric properties, optical transparency, low Young’s modulus and softness, ISO 10993 certified biocompatibility and biostability, as well as good barrier properties against water vapor or chemicals and good temperature stability in comparison with other polymers [1,2,3,4]. Due to these properties, Parylene is compatible with most microtechnologies. Furthermore, Parylene is deposited from the gas phase at room temperature, resulting in highly conformal coatings without internal stresses [3,5].

Traditionally, Parylene is used for the encapsulation of medical implants or electronics [4,6,7,8,9,10,11]. However, in the last years, new applications in the field of MEMS also have been established using Parylene as a functional or structural material, e.g., as an adhesive for wafer and chip bonding [1,12,13]. MEMS using Parylene include not only acceleration sensors [14], pressure and force sensors [15,16], acoustics [17,18], optical devices [19,20], microfluidics [21,22], and lab on chip [23], but also its use as a substrate material for flexible electronics [24,25]. The increased usage of Parylene for the various applications raises the question regarding the reliability and lifetime of Parylene, i.e., any impacts of aging on its properties.

Existing studies are performed in the context of medical applications and the barrier properties of Parylene and based on accelerated aging [26,27,28,29,30,31,32,33,34]. Doing so, the aging of Parylene is accelerated by elevated temperatures to determine any impact on chemical or physical properties [35]. The accelerated aging can be converted into equivalent non-accelerated aging at ambient or body temperature by the “10-degree rule”. This rule is derived from an Arrhenius function (refer to Appendix A for more details) and states that for every temperature increase of 10 K the reaction and aging rate will double [6,36,37].

Based on this rule, e.g., the aging behavior of Parylene C layers and composites based on Al_2_O_3_ deposited by atomic layer deposition (ALD) and Parylene C is studied at temperatures of 37 °C, 57 °C, 60 °C, 67 °C, 80 °C, and 87 °C for up to 260 days while soaking in phosphate buffered solution (PBS). All samples proved a good stability of Parylene C and an enhanced stability for the composites [26,27,28,29,30,31,38]. Other studies compare Parylene implanted for 3.25 years and acceleratingly aged at 67 °C and 87 °C in PBS with added hydrogen peroxide to simulate a reactive environment. The latter is proven to have a negative impact on Parylene C [32,33]. Only a very limited number of studies based on non-accelerated aging at ambient temperatures for 276 days and a year, respectively, are published. These studies show that Parylene C is stable. However, the characterization was limited to electrical quantities, and the studies are based again on soaking in PBS [39,40]. Finally, accelerated aging at higher temperatures between 125 °C and 200 °C is proven to oxidize Parylene C, predicting 130,000 years of lifetime for Parylene C [41].

The aging mechanisms of Parylene are supposed to be based on the formation of carbonyl groups due to the formation of ester bonds at aliphatic Parylene bonds. This causes chain scission and further reaction with oxygen. The impact on the mechanical properties is embrittlement and the reduction in the tensile strength, as well as crack formation. Another mechanism discussed in the literature is the photocatalytic cleavage of chlorine that leads to the creation of radical sites in chains and, hence, intramolecular phenylation and hydrogen abstraction [5,32,42,43].

Due to the limited temperature stability of Parylene as well as the complex reaction kinetics causing various and partially unknown aging mechanisms, it is doubtful whether the “10-degree rule” can be applied without limitations. Within this study, the first long-term and non-accelerated aging of Parylene C is performed for five years (~1800 days) of aging in ambient temperature. With respect of the usage of Parylene as a material for MEMS and substrate for flexible electronics, a non-reactive environment is chosen for aging. Doing so, the barrier properties, optical properties, and the chemical stability are investigated in dependency of different post-treatments of Parylene C and non-accelerated aging. Additionally, the mechanical properties of Parylene C are investigated in comparison for non-accelerated and accelerated aged Parylene C.

## 2. Materials and Methods

### 2.1. Sample Preparation for the Investigation of the Impact of Aging on the Barrier Properties, the Chemical Stability, and the Optical Properties of Parylene C

**Figure 1:**
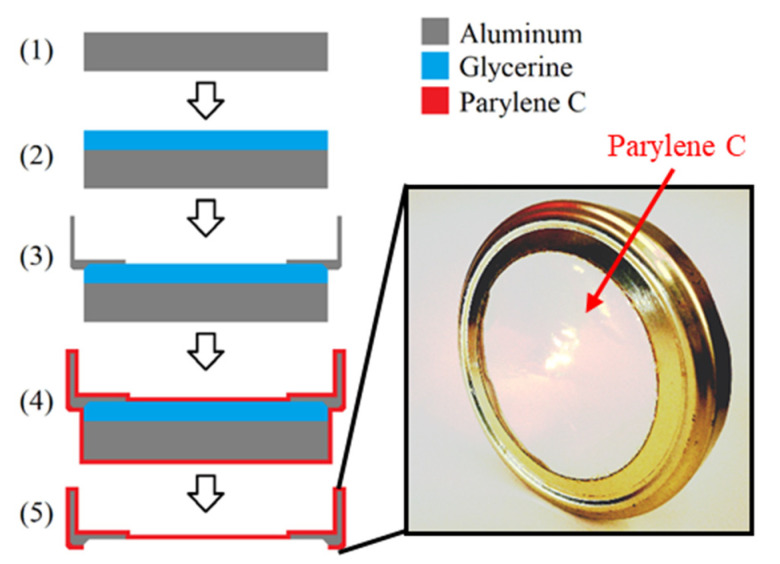
Fabrication process of large free-standing Parylene C membranes for the investigation of the barrier properties, the optical properties, and the chemical bondings.

**Figure 2:**
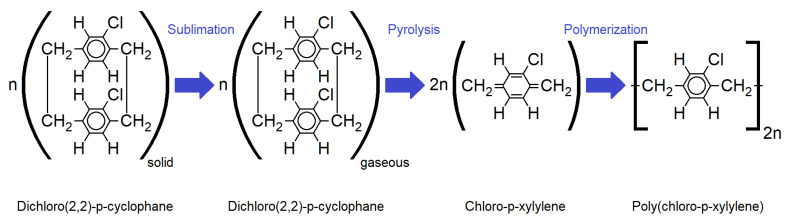
Molecular structure for each step of the Gorham CVD process for the deposition of Parylene C [2].

For the investigation of the impact of aging on the barrier properties, the chemical stability and the optical properties of Parylene C, large-scale free-standing circular membranes with a diameter d of 8.1 cm, were fabricated. The fabrication process is depicted in Figure 1 and explained in detail elsewhere [3]. It can be summarized by the following five steps: On an aluminum plate of ~9 cm diameter (1) liquid glycerine (Brenntag GmbH, Germany) was spin-coated at 1000 rpm for 10 s (SpinCoater CT62, SÜSS MicroTech AG, Germany). Next, a cap for a regular screw-top jar that has a hole for the Parylene membrane to be fabricated was placed on the glycerine-coated aluminum plate (3). Subsequently to this, the sample was coated with Parylene C by chemical vapor deposition (CVD) in accordance with the Gorham process depicted in Figure 2.

Doing so, first, a solid dimer (Plasma Parylene Systems GmbH, Germany) was sublimed at 130 °C. Second, the obtained gaseous dimer was thermally cracked into reactive monomers by pyrolysis at 740 °C. In the third step, the monomers react to linear polymer chains at room temperature. The process was performed in vacuum at pressures <5.5 Pa. For the fabrication of the samples, a Plasma Parylene LC 300 RW (Plasma Parylene Systems GmbH, Germany) was used.

In the final step of the sample fabrication, the Parylene was cut on the bottom side of the sample by a scalpel, so that the cap gets released from the aluminum substrate by the glycerine to form a free-standing Parylene membrane (5). In order to remove excess glycerine from the Parylene and the cap, the samples were stored in deionized water overnight.

For sample preparation, the Parylene thickness, the deposition rate, and sublimation temperature, respectively, as well as the post-treatment, were systematically varied. An overview of the different samples is given in Table 1. Due to statistic and redundancy reasons, for all parameter variations listed in Table 1, two identical samples were fabricated, that were investigated in parallel.

For all samples with a thickness below 3 µm, a dimer mass of 5 g was used. The thicker Parylene membranes were obtained by increasing the dimer mass to 10 g and 15 g, respectively. A regular process was performed with a sublimation temperature of 130 °C, since this was the optimized temperature for the deposition of high-quality Parylene C in a short process time [3]. For one sample, a slow Parylene C deposition was performed by using a sublimation temperature of 100 °C only, in order to investigate any potential improvement of the barrier properties. The thicknesses of the Parylene membranes given in Table 1 were measured by profilometry (Alpha-Step 500, KLA Tencor GmbH, Germany) and reflectometry (F20, Filmetrics Europe GmbH, Germany) on a silicon chip coated in parallel. Furthermore, IR spectroscopy was used to determine the thickness (see Section 2.4).

### 2.2. Post-Treatment and Aging

Furthermore, an application relevant post-processing treatments was applied in order to investigate, whether they impair the change in Parylene C properties for the short term or long term. These post-treatments are summarized in Table 1 as well. With respect to medical applications, steam sterilization with saturated steam of deionized water at 121 °C for 20 min an autoclave (DX-23, Systec GmbH) and electron beam sterilization according to DIN EN ISO 11137-1:2013-12 and DIN EN ISO 11137-2:2013-09 using a dose of at least 25 kGy were used.

Considering the usage of Parylene as a material for MEMS, annealing in air at 200 °C for 15 min to simulate any thermal budget caused by microfabrication processes, as well as a treatment in UV light with a wavelength of 254 nm and a power of 40 W for 30 min to simulate extreme conditions of lithography exposure was performed. The temperature was chosen, since it is the maximum temperature that Parylene C can endure for the short term in air [44].

To investigate the impact of aging on the Parylene C barrier properties, the chemical stability, optical properties, the water vapor transmission rate (see Section 2.3), as well as UV/VIS and IR spectra (see Section 2.4) were measured directly after fabrication, as well as after ~600 days, ~1200 days, and ~1800 days of non-accelerated aging. All samples were stored in a clean room for non-accelerated aging at 22 °C and 50 %RH (relative humidity).

### 2.3. Water Vapor Transmission Rate

**Figure 3:**
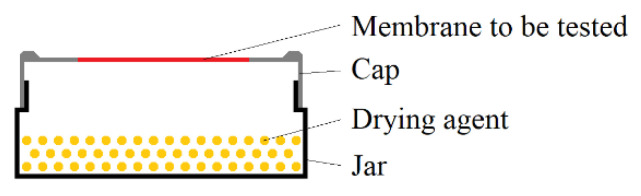
Sample setup for the determination of the WVTR according to DIN 53122.

For the evaluation of the barrier properties of Parylene, the water vapor transmission rate (WVTR) was measured in accordance to DIN 53122. This DIN describes a gravimetric method for the determination of the WVTR using samples, which are schematically depicted in Figure 3, and which allow the application of a humidity gradient over a membrane material of interest. These samples were stored in a defined climate with high humidity for a certain time. The drying agent inside the sample absorbed water that permeated through the membrane to keep a relative humidity of 0 %RH inside the sample. Hence, the sample mass increased over time.

To realize the samples according to Figure 3, 100 g of pre-dried (for >2 h at 135 °C in air) silica gel (Engelhard Process Chemicals GmbH, Germany) was used as a drying agent (water absorption capacity: 36 g per 100 g silica gel) and filled into the glasses. The caps with the free-standing Parylene C membrane were screwed on the glasses filled with silica gel and sealed with gum paste (P.C. Flex Mask, smartTec GmbH, Germany) and self-vulcanizing rubber tape (Würth GmbH & Co. KG, Germany). For the measurements of the WVTR of Parylene C, the samples were placed in a climate chamber (CSR-60/600-5, CTS Clima Temperatur Systeme GmbH, Germany) with climate B of DIN 53,122 (38 °C and 90 %RH). The mass of each sample was determined daily over the storage time Δt of at least 14 days.

The WVTR finally was calculated according to Equation (1) from the mass increase Δm by normalizing it on storage time Δt, the thickness of the Parylene membrane Δs, as well as the membrane area (membrane diameter d). To consider the leakage rate of the samples, i.e., the absorbed water that permeates through the sealing instead of the Parylene membrane, the mass increase Δm of two reference samples with a closed cap (no Parylene membrane) was measured in parallel and subtracted from the mass increase Δm in the other samples.

(1) $$\mathrm{WVTR}=\frac{\;\Delta m\;\cdot \;\Delta s\;}{\frac{\pi }{4}\;\cdot {\;d}^{2}\;\cdot \;\Delta t}$$

### 2.4. Spectroscopy

For the investigation of the impact of aging on the chemical stability of Parylene C and in particular on its oxidation, the free-standing membranes were measured by Fourier-transform infrared spectroscopy (FTIR) using a VERTEX 70 FTIR spectrometer (Bruker Corporation, MA, USA) in the range of 3300 cm^−1^ to 700 cm^−1^. The FTIR spectrum of the background was measured separately and subtracted from the spectra of each sample. All FTIR measurements were performed in transmission through the Parylene membranes.

Doing so, constructive and destructive interference caused the overlap of the characteristic FTIR absorption bands of the obtained spectra with a sinusoidal shape. The requirement for constructive interference is given in Equation (2) with the wavelength λ, the refractive index *n*, the Parylene thickness s, and the integer i.

(2) $$\lambda =2\;\cdot \;\frac{n\;\cdot \;s}{i}$$

Table A1 in Appendix B compares the calculated Parylene thicknesses according to Equation (2), using the assumption of constructive interference at the observed sinus maxima, with the values given in Table 1. Only small differences prove that the sinusoidal shape was indeed caused by interferences due to measurement setup in transmission.

**Figure A1:**
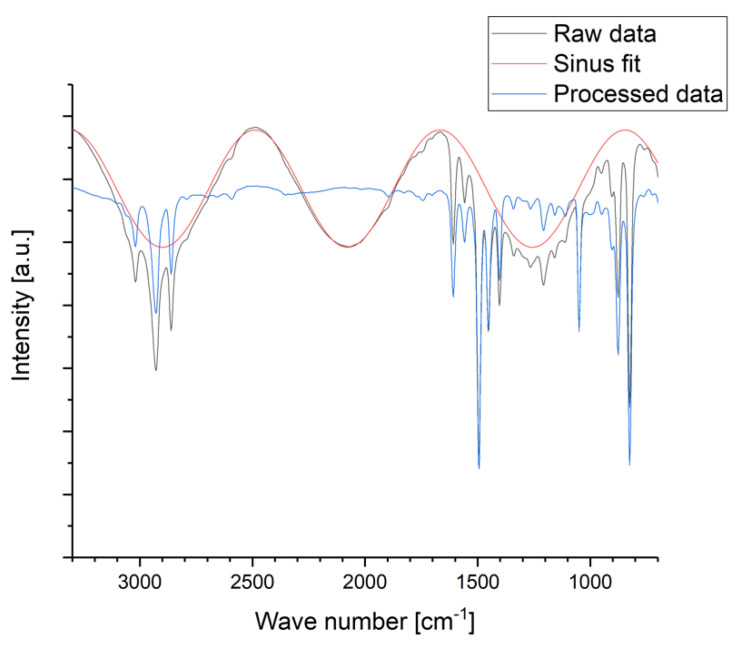
Example for the post-processing of the measured FTIR raw data to smooth the spectra: The raw data are subtracted by a fitted sinus function to obtain the processed data.

In order to remove the sinusoidal shape from the FTIR spectra, the obtained raw data were post-processed by manual fitting of a sinus function for the signal intensity I [a.u.] in dependence of the wave number k [cm^−1^] with the general parameters of a sinus function A [a.u.], B [cm], C [a.u.], and D [a.u.] according to Equation (3). The fitted sinus function was subtracted from each spectrum as depicted in Figure A1 in Appendix B.

(3) $$I\left(k\right)=I\;\left(\frac{1}{\lambda }\right)=A\;\cdot \;\sin\;\left(B\;\cdot \;k+C\right)+D$$

**Figure A2:**
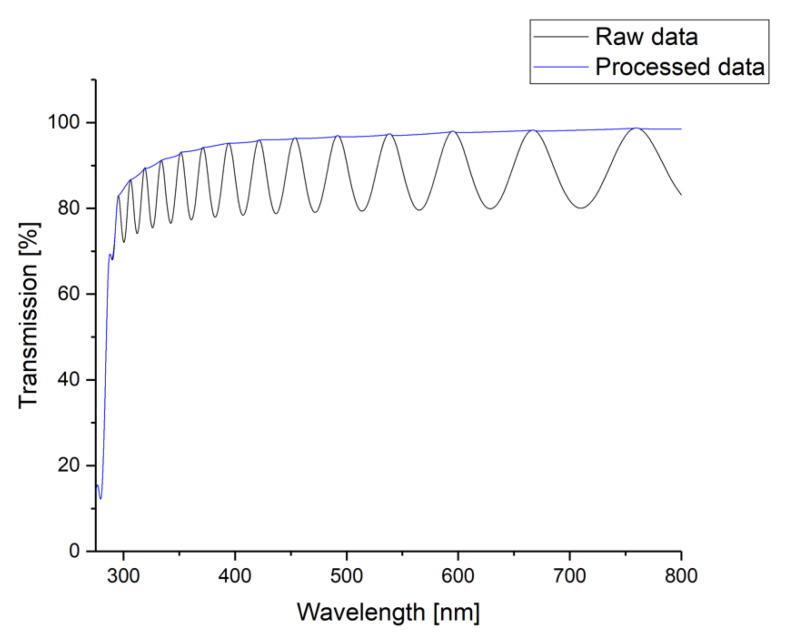
Example for the post-processing of the measured UV-VIS raw data to smooth the spectra: The maxima of the raw data, i.e., the constructive interferences are fitted.

The impact of aging processes on the optical properties of Parlyene was investigated by UV/VIS spectroscopy using an UviLine 9400 (SCHOTT Instruments, Germany) and a UV-3100PC Spectrophotometer (VWR International GmbH, Germany), respectively. The measured wavelength range was 275 nm to 800 nm. The measurements were performed in transmission again. Hence, the overlap of the obtained spectrum with a sinusoidal shape caused by constructive and destructive interferences was observed again. The obtained sinusoidal shape shows an irregular period and, thus, was impossible to fit by a regular sinus function. Therefore, the obtained spectra were flattened by fitting the maxima, i.e., the points of constructive interference only, as it is depicted in Figure A2 in Appendix B.

### 2.5. Sample Preparation for the Investigation of the Mechanical Properties of Parylene C

For the investigation of the mechanical properties of Parylene C, bare silicon chips obtained from 6” wafers were coated accordingly with the Gorham process described above, using a sublimation temperature of 130 °C. The Young’s modulus as well as the indentation hardness were measured before and after the end of the non-accelerated aging (~1800 days, respectively) using nano-indentation. Additionally, these samples are compared with accelerated aged samples. Doing so, the Parylene samples were annealed in air at 87 °C in an oven (Memmert GmbH & Co. KG, Germany) for 97 h, 291 h, and 472 h to realize samples that are equivalent to non-accelerated aging for 1, 3, and 5 years as calculated by the “10-degree rule”. An annealing temperature of 87 °C was chosen since this temperature is the maximum temperature found in the literature about thermally accelerated polymer aging [6,32,33].

In order to investigate the impact of a thermal budget on Parylene C in comparison to the impact of aging, two extra samples were added, which were treated using a typical microfabrication thermal process. An annealing process at 115 °C for 100 h in air was chosen to represent the temperature of a typical sinter process, e.g., for the sintering of aerosol jet printed silver inks using an extended time to maximize the potentially observed impact [45,46]. In order to test whether a limit in similarity to a saturation can be observed for the impact of aging or annealing on the mechanical properties of Parylene C, the non-accelerated aged sample was additionally annealed applying the parameters of the sinter process mentioned above. Table 2 summarizes all samples on which the mechanical properties were measured.

### 2.6. Mechanical Characterisation by Nano-Indentation

The Young’s modulus E and the indentation hardness H were investigated by continuous stiffness measurement (CSM) for thin films via nano-indentation. The method is described in detail elsewhere [47] and briefly summarized in Appendix C. Note that the obtained results of the indentation hardness cannot be converted into the established mechanical hardness methods, such as Vickers hardness or Brinell hardness. However, they are still representing the resistance of the Parylene against localized plastic deformation.

**Figure 4:**
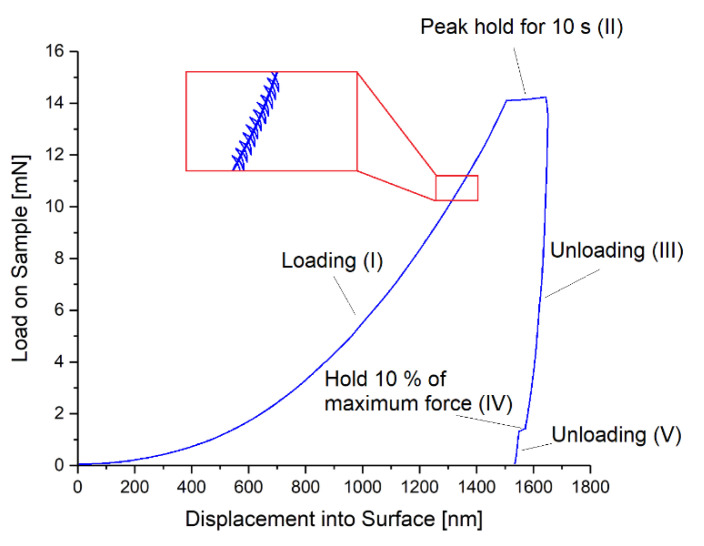
Applied load cycle for the nano-indentation measurements consisting of five parts: (I) increasing the load, (II) holding the maximum force, (III) decreasing the load until 10% of maximum force, (IV) holding 10% of the maximum force for 75 s, and (V) decreasing the load until zero.

In preparation of the measurements, the Parylene C coated chips of about 2 × 2 cm^2^ size were glued on a glass slide using super glue. For the nano-indentation measurements a G200 nano-indenter (Agilent Technologies Inc., CA, USA) with an XP load head and a Berkovich tip (calibrated on fused silica for 2000 nm penetration depth) was used. The applied load cycle on the tip for each measurement is depicted in Figure 4 and consists of five parts: (I) increasing the load, (II) holding the maximum force for 10 s, (III) decreasing the load until 10% of maximum force, (IV) holding 10% of the maximum force for 75 s (drift correction), and (V) decreasing the load until zero. During the measurement, a depth limit of 1500 nm, a strain rate of 0.05 s^−1^, and a peak hold time of 10 s were applied. The Poisson’s ratios of Parylene C and the silicon substrate were assumed to be 0.4 [48] and 0.3 [49], respectively. The Young’s modulus of the silicon substrate was 170 GPa [50], which was also verified by indentation on the silicon substrate only. Thin film and drift corrections were applied as described in Appendix C. For averaging, 25 indents were performed for each sample and a range of 9.5–10.5% of the film thickness was considered for analysis. For microscopic imaging, a regular light microscope Eclipse L200 (Nikon Corporation, Japan) was used.

## 3. Results

### 3.1. Impacts of Aging on the Barrier Properties

**Figure 5:**
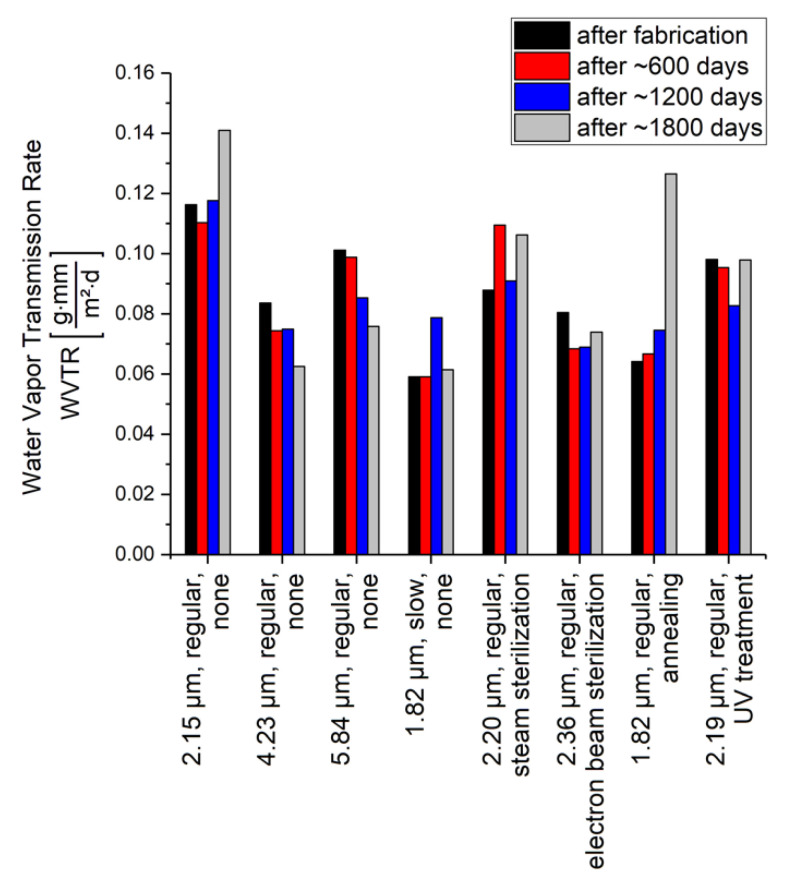
Results of water vapor transmission rate WVTR for the different samples and after fabrication as well as after ~600 days, ~1200 days, and ~1800 days of non-accelerated aging.

**Figure A4:**
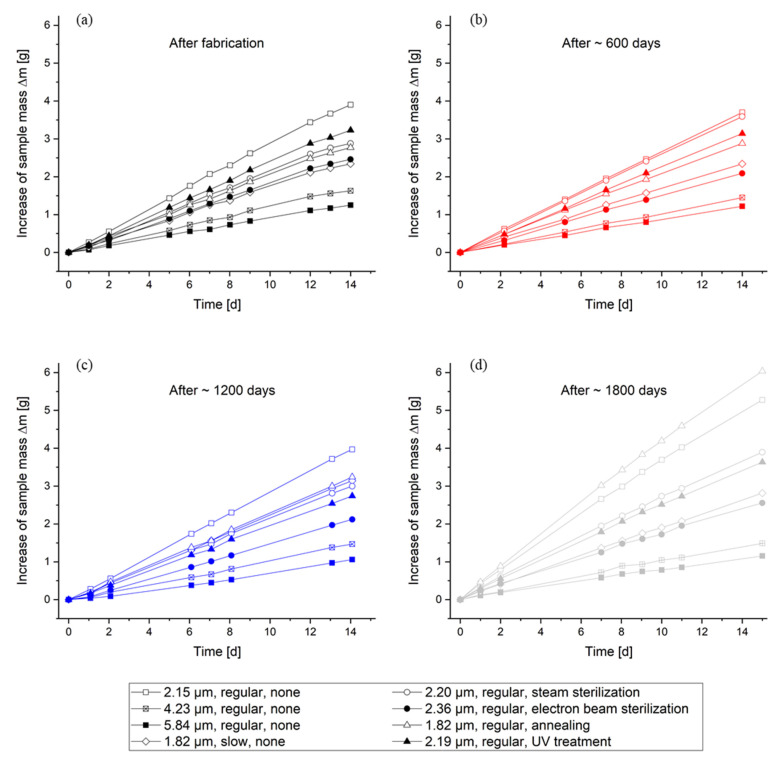
Time-dependent increase in the sample mass during the gravimetric determination of the WVTR according to DIN 53122 after fabrication (**a**) and non-accelerated aging of ~600 days (**b**), ~1200 days (**c**), and ~1800 days (**d**), respectively.

For all WVTR measurements, the increase in the sample masses over time are depicted in Figure A4 in Appendix D and show a linear dependency and absorbed water masses up to 7 g. Hence, it can be concluded that the limit of the water absorption capacity of the silica gel is not reached, and the complete measurement period can be used for the calculation of the WVTR. The results of the calculated WVTR are depicted in Figure 5 for all samples of Table 1 and for the different durations of non-accelerated aging.

For all samples, the WVTR is in or very close to the typical range of 0.06 $\frac{g\cdot \mathrm{mm}}{m^{2}\cdot d}$ to 0.08 $\frac{g\cdot \mathrm{mm}}{m^{2}\cdot d}$ given in the literature [44,51], whereas in particular for the used test climate of 38 °C and 90 %RH, the higher value is reported [51]. Since the WVTR is normalized on the thickness of the tested membrane according to Equation (1), the three samples that are fabricated by a regular deposition process and without any post-treatment can be used to estimate an accuracy of the determined WVTR of >0.03 $\frac{g\cdot \mathrm{mm}}{m^{2}\cdot d}$. The slight decrease in the WVTR over aging time suggested by the regularly deposited samples with Parylene thicknesses >3 µm and without any post-treatment would be in contrast to the thinner sample of 2.15 µm thickness. For Parylene C, which is fabricated by a slower deposition process, a decreased WVTR could be slightly indicated. However, this improvement of the barrier properties would be less than the accuracy of the test method.

Comparing the measurement results after fabrication, it can be concluded that none of the applied post-treatments has an impact on the WVTR of Parylene C. Furthermore, the results depicted in Figure 5 show that no impact of non-accelerated aging on the WVTR of Parylene C can be observed. The deviations in the WVTR over time are less than the accuracy of the method, except of the annealed sample.

For the annealed sample, a high increase in the WVTR of >65% between ~1200 days and ~1800 days of aging is observed. However, it has to be noted that the Parylene membrane of these samples were in high mechanical tension, probably due to the reorganization and increased crystallinity caused by the annealing and reported in the literature [52]. During storage in the climate chamber for the measurement after ~1800 days, the Parylene membrane of one sample even ruptured. Hence, it is very likely that the second sample, depicted in Figure 5, also shows cracks or defects on a microscale, which would increase the WVTR. Considering that the thinnest untreated Parylene membrane has a similar membrane thickness compared to the annealed sample but does not show an increase in the WVTR over time in the same dimension, it can be concluded that the effect is particularly caused by the annealing. If defects on the microscale would be caused by annealing, this would be a limitation to be considered when performing accelerated aging.

In summary, the barrier properties of Parylene C are not affected by non-accelerated aging. Furthermore, this result is independent of post-treatment, such as sterilization or UV-treatment. Even though annealing as post-treatment does not seem to affect either the initial barrier properties or their non-accelerated aging behavior, a more detailed investigation is required to determine how the barrier properties are related to defects induced by annealing. Additionally, a reduced deposition rate of Parylene C does not cause any improvement with respect to the WVTR or the aging behavior of Parylene C.

### 3.2. Impacts of Aging on Chemical Stability

**Figure 6:**
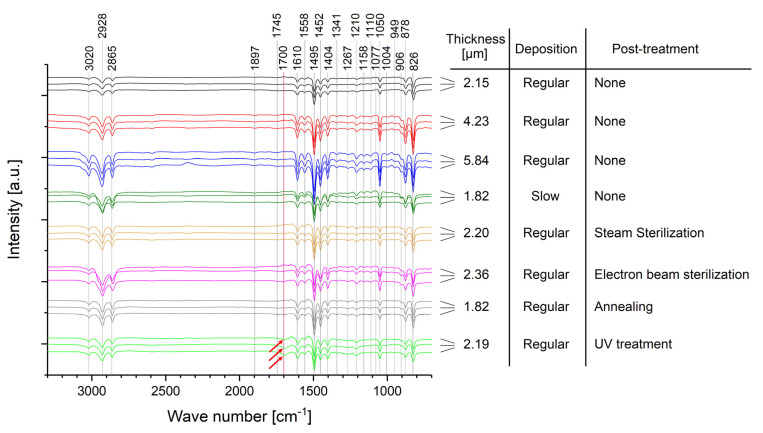
FTIR spectra for the different samples after correction of the sinusoidal overlap. Each sample is represented by three spectra obtained after fabrication (upper) and after a non-accelerated aging time of ~1200 days (middle) and ~1800 days (lower). The red arrows highlight the oxidation peaks measured for the UV post-treated samples.

The measured FTIR spectra are depicted in Figure 6 and show the typical bands according to the literature [32,42,51,53,54,55]. The detailed assignment of the wave numbers to the vibrational modes are summarized in Table A2 in Appendix E.

Wave numbers above 3000 cm^−1^ (3020 cm^−1^) are assigned to aromatic, whereas wave numbers below 3000 cm^−1^ (2865 cm^−1^, 2928 cm^−1^) are assigned to aliphatic C−H stretching vibrations [56,57]. However, since the wave number of 3020 cm^−1^ is very close to the 3000 cm^−1^ limit, other scientific literature assigns it to C−H aliphatic stretching as well [42,53]. Wave numbers of 1897 cm^−1^, 1745 cm^−1^, 949 cm^−1^, 906 cm^−1^, and 878 cm^−1^ are assigned with out-of-plane wagging of C−H bonds [42,53,56]. The peak at 1700 cm^−1^ can be observed due to oxidation of Parylene C and represents the formation of carbonyl bonds [32,42,53]. C=C stretching vibrations in the aromatic ring are assigned to 1558 cm^−1^ and 1610 cm^−1^, whereas semicircle stretching is assigned to 1341 cm^−1^, 1404 cm^−1^, and 1495 cm^−1^, respectively [42,53]. Several absorptions bands are assigned to the *p*-CH_2_ groups on the aromatic ring: 1077 cm^−1^, 1110 cm^−1^, 1158 cm^−1^, 1210 cm^−1^, and 1267 cm^−1^ are caused by wagging [42,53], whereas 1452 cm^−1^ can be assigned either to CH_2_ deformation [42,53] or to the bonding of this group to the ring [56]. C−C aromatic and aliphatic stretching vibrations are assigned to 1493 cm^−1^ and 826 cm^−1^, respectively [57]. However, the band at 826 cm^−1^ can also be assigned to two neighboring hydrogen atoms bonded to the ring [56]. Finally, C−H groups can be observed at 826 cm^−1^ with out-of-plane wagging or at 1004 cm^−1^ and 1050 cm^−1^ with in-plane bending [42,53]. Additionally, the absorption band at 1050 cm^−1^ is characteristic for the chlorine bonded to the aromatic ring in Parylene C [56].

In conclusion of the spectra given in Figure 6, no difference is observed for all samples without post-treatment and, hence, no impact of non-accelerated aging up to ~1800 days. This is independent of the Parylene thickness and whether the Parylene is deposited by a regular or a slow process (130 °C and 100 °C sublimation temperature, respectively). These samples show an increase in the peak intensity with increasing thickness of the Parylene membrane due to more material transmitted by the IR laser beam.

Furthermore, neither the two tested sterilization methods nor the annealing post-treatment alter the chemical composition of Parylene C, nor do they cause any negative long-term impact on the aging behavior of Parylene C. However, for a sample that was post-treated by UV radiation, a small oxidation peak is observed directly after the fabrication of the sample. Additionally, this oxidation peak slightly increases during non-accelerated aging. Hence, the chemical composition of Parylene C is changed in the short term and with a negative long-term impact due to UV-induced oxidation. This impact is important for the integration of Parylene C into MEMS considering UV exposure due to lithography. Based on this, lithography under inert conditions could be advantageous. In conclusion, besides the negative impact of UV radiation on its long-term stability, Parylene C shows an excellent stability of its chemical composition against the tested post-treatments and against non-accelerated aging over at least around five years.

### 3.3. Impacts of Aging on the Optical Properties

**Figure 7:**
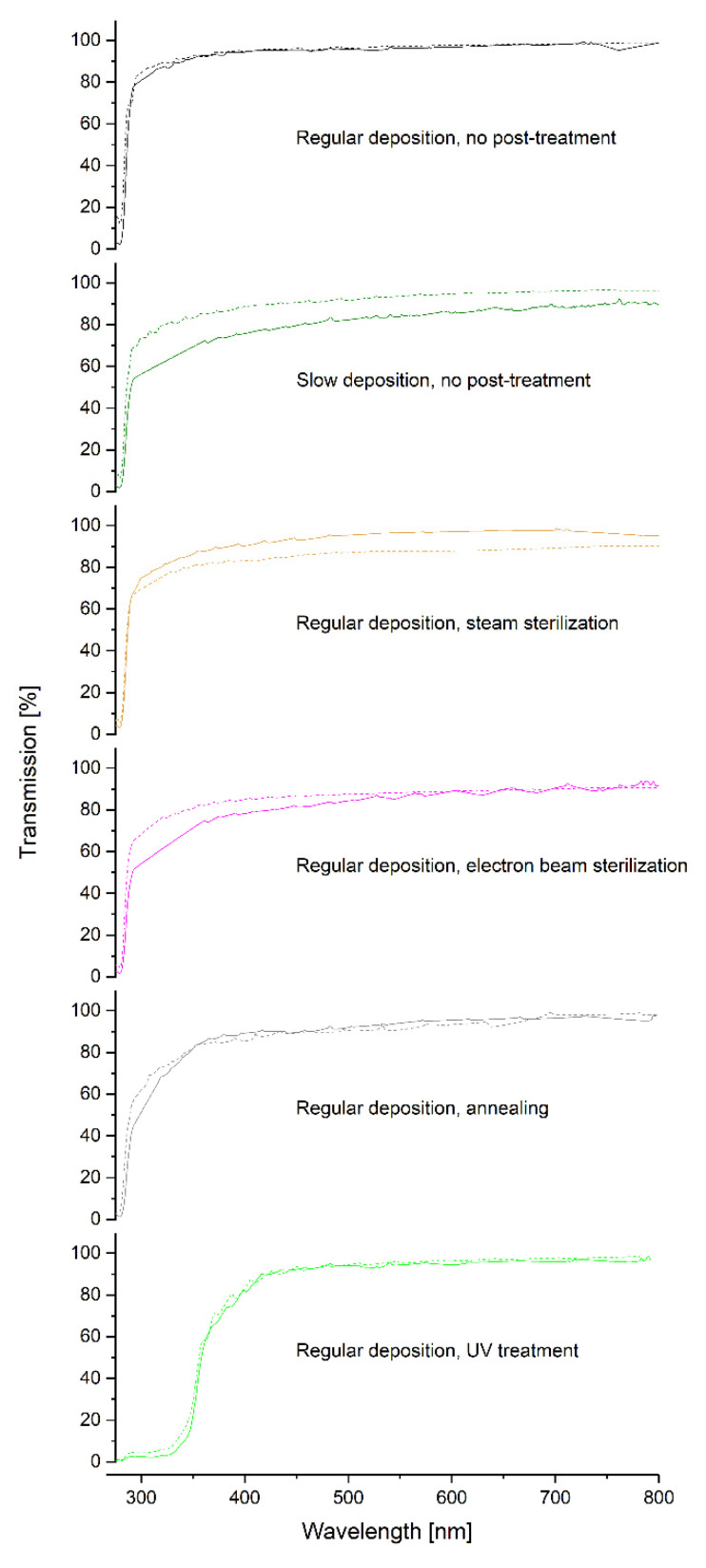
UV-VIS spectra for the different samples after correction of the sinusoidal overlap. The solid lines represent the spectra after fabrication, whereas the dashed lines represent the spectra after a non-accelerated aging time of ~1800 days.

The obtained and post-processed UV-VIS spectra are depicted in Figure 7. The spectra of the samples with thickness variation only are not considered, since no deviation from the spectrum measured at the sample, which was deposited regularly and received no post-treatment, is expected. Due to the low dose during the measurements, it is unlikely that the samples will be altered by the UV-VIS measurement itself. Furthermore, and in case an alteration takes place during the measurements, due to the small spot size of the beam used in comparison with the large membrane, it is unlikely that during the different measurements performed on one sample at different times that the same sample position is measured twice.

Note that due to the measurement setup, the samples are not perpendicular to the beam path of the spectrometer. Hence, the fraction of reflection and absorption is increased due to a longer penetration path of the beam. In particular, since the angle between the sample and the beam path is slightly different for each measurement, a direct comparison of the absolute transmission percentage is not possible. This is also indicated by the fact that some samples show initially a higher transmission compared to their spectra after non-accelerated aging, whereas for other samples, the opposite tendency is observed. Nevertheless, all samples have a high transmission of ≥80% over the whole visible wavelength range, most even ≥90%. This is independent of the post-treatment and also of aging.

However, a direct comparison of the cutoff in the UV range is possible. This cutoff is in the range of 285 nm to 290 nm (50% absorption) for all samples except the UV post-treated Parylene C. Hence, neither sterilization treatments nor annealing or a slowed Parylene C deposition have any impact on the spectra in the visible wavelength range. In contrast to this, for a UV-treatment, a shift of the absorption bandto values between 355 nm and 360 nm (50% absorption) is observed, even though the samples appear optically to be as transparent as the other samples. This impact of UV treatments has to be considered when machining Parylene C with UV exposing processes, such as lithography. Additionally, this effect could be intentionally used for processes, such as selective laser ablation, since the wavelength of the used laser should be below the absorption band for the ablation of Parylene using a UV laser, e.g., with a wavelength of 266 nm. However, these lasers are expensive and have a short lifetime only due to high deterioration. Shifting the absorption band to values between 355 nm and 360 nm by a UV post-treatment could enable the usage of a laser with a higher wavelength, e.g., 355 nm. Still, it needs to be investigated in detail, whether the Parylene C is just altered on its surface only or in its bulk. The additional oxidation peak of the UV-treated Parylene C in the FTIR spectrum depicted in Figure 6 could be an indication for an alteration of the Parylene C bulk properties since an alteration that would be limited to the surface properties would cause an almost negligible oxidation peak in the FTIR spectrum. Furthermore, the tunability of the shift of the absorption band by varying the UV intensity or exposing duration of the UV post-treatment needs to be the subject of a more detailed investigation.

Finally, for all samples, independent of their post-treatments, no impact of non-accelerated aging over ~1800 days on the optical properties is observed.

### 3.4. Impacts of Aging on the Mechanical Properties

**Figure 8:**
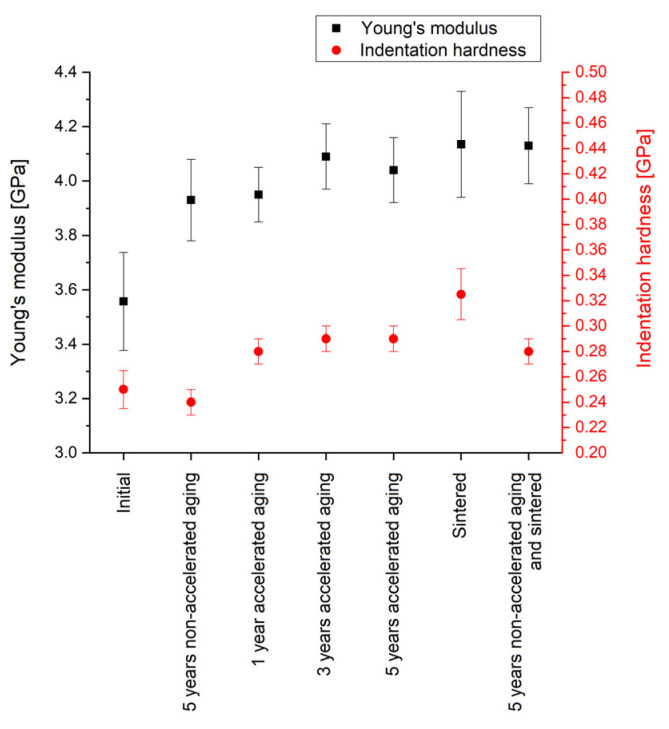
Results of the nano-indentation measurements for the Young’s modulus and the indentation hardness of Parylene C. The error bars represent the standard deviation.

Figure 8 summarizes the results of the nano-indentation measurements. The obtained Young’s modulus of 3.56 GPa of Parylene C as deposited (initial) shows a deviation of ~10% to the value of 3.20 GPa given in the literature [44]. Hence, and in consideration that the deviation of the Young’s modulus measured by nano-indentation and tensile test can be up to 20% due to measuring on thin films and the influence of the rigid substrate, the obtained values are in good accordance with the literature. Even though the values for the indentation hardness cannot be compared with other methods for mechanical hardness, such as Vickers or Brinell hardness, the low values for both, the indentation hardness, and the Young’s modulus indicate the softness of Parylene.

**Figure 9:**
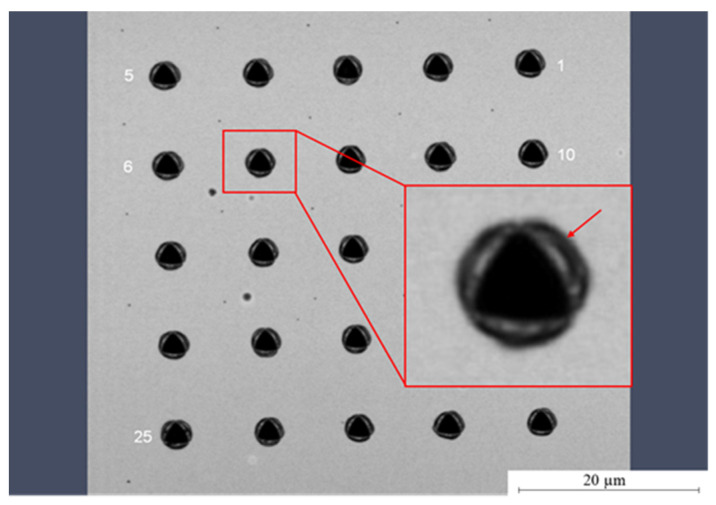
Light microscopic image of the nano-indents on Parylene C. The typical pile-ups (arrow) observed for soft materials is clearly visible.

The typical bulges, called pile-ups, depicted in Figure 9 that can be observed after nano-indentation are an additional feature for soft materials. This softness of Parylene C is advantageous when using the material as a substrate for flexible electronics and when integrating it into MEMS for the realization of highly sensitive sensors and actuators. Note that the pile-ups are not considered quantitatively for the evaluation.

Five years of non-accelerated aging increase the Young’s modulus significantly by 10% to a value of 3.93 GPa; however, the indentation hardness is not altered. Compared to this, five years of accelerated aging cause a similar increase in the Young’s modulus to 4.04 GPa; however, in contrast to non-accelerated aging, the indentation hardness is increased as well. This is in accordance with the altering of the mechanical properties at elevated temperatures described in the literature [53]. Additionally, it is interesting to note that also the samples of free-standing Parylene membranes used for the WVTR measurements showed a more stressed membrane after annealing at 200 °C.

However, it is still noteworthy that within our experiments the material alteration can be observed for annealing temperatures lower than the temperature for long-term exposure of 125 °C given in the literature [44]. The increase in the indentation hardness could be due to recrystallization processes caused by increased temperatures and leading to a higher share of crystalline material in the regularly partly crystalline polymer [52]. In contrast to that, an impact on the unit cell of the crystalline domains is unlikely. For the different durations of accelerated aging, neither for the Young’s modulus nor for the indentation hardness a time dependency is observed.

In comparison with the impact of aging, for annealing a slightly higher increase in the Young’s modulus and a significantly increase in the indentation hardness are observed. This effect has to be considered when processing Parylene C by microtechnologies. Interestingly, the sample that is aged and sintered subsequently shows no further increase in the Young’s modulus and a lower indentation hardness compared with the sample that is sintered after deposition. This could suggest either a kind of saturation of the Young’s modulus or a reduced impact of elevated temperatures on (non-accelerated) aged Parylene C.

In summary, the nano-indentation measurements prove that aging has only a small impact on the mechanical properties of Parylene C. Furthermore, for the chosen parameters, the model of accelerated aging is not in accordance with non-accelerated aging. In contrast to the indentation hardness that depends only on the impact of temperature, the Young’s modulus additionally depends on the age of the Parylene C. Furthermore, the method of accelerated aging by increased temperature is only suitable for testing the mechanical properties of Parylene C within proper limits and not for all mechanical quantities.

### 3.5. Summary

Table 3 summarizes the investigated properties of Parylene C with respect to their dependency on post-treatments and/or non-accelerated aging. Considering that accelerated aging is induced by heat treatment, the former was considered as a “post-treatment” for the classifications within this table.

Table 3 suggests the classification of the investigated properties into the three following groups considering their dependency on post-treatments and/or non-accelerated aging:

1. The property neither depends on a post-treatment nor on non-accelerated aging.
2. The property depends on the post-treatment but not on non-accelerated aging.
3. The property depends on different post-treatments and on non-accelerated aging.

## 4. Discussion

The results produced in this study show that the non-accelerated aging of Parylene C does not cause any changes with respect to barrier properties, optical properties, and chemical composition for a time frame of five years. Hence, this study supports a vast majority of the scientific literature, which reports the usage of Parylene for encapsulation, e.g., of medical implants, for its usage in MEMS, electronic components, or sensors as well as a material for flexible electronics. Particularly, the results prove Parylene to be a highly reliable material for these applications. At the same time, the results of this study confirm the compatibility of Parylene C with most of the standard microtechnologies, even though particular attention has to be drawn on all processes that include UV and thermal annealing, respectively. The results suggest that UV induces photo-oxidation of Parylene C in the bulk material, which changes particularly the optical properties. This impact has to be considered for outdoor applications but can be advantageous for tailoring the absorption of Parylene C as well.

Within this study, the FTIR spectroscopy was successfully used in transmission on ultra-thin, free-standing films, and a related post-processing data were established, which was beyond standard procedures.

In contrast to the barrier properties, optical properties and chemical stability, the mechanical properties of Parylene C will be altered by aging, i.e., the Young’s modulus increases. These alterations caused by aging are not critical for most applications of Parylene. However, they are still noteworthy and should be in a particular focus when using Parylene as a free-standing material for MEMS applications. It remains to be seen that the mechanical alterations can be observed even at temperatures lower than the temperature limit given for long-term stability in the datasheet for Parylene C [44].

Furthermore, differences between non-accelerated aging and accelerated aging with respect to its impact on different mechanical properties are observed. Both aging methods increase the Young’s modulus; however, the hardness is only increased by accelerated aging. The results indicate that the observed increase in the hardness is caused by the thermal budget that is connected to the accelerated aging and the required activation energy given in the Arrhenius equation (Equation (A1)), respectively. Moreover, the results indicate a dependency of the hardness increases on the chosen temperature and also the point in time of the thermal treatment. Previous studies determined recrystallization processes in Parylene to be caused by thermal annealing. It can be concluded, in contrast to non-acclerated aging, that accelerated aging will enforce recrystallization processes due to its nature of being a thermal process. Thus, the use of accelerated aging has to be carefully evaluated with respect to avoid misleading conclusions to the reality and non-accelerated aging, respectively. Similar effects might be noticeable also for other polymers.

Based on previous studies and the new results obtained within this work, for the aging mechanism in Parylene C, the following assumptions can be derived:

- The hardness depends significantly on the annealing temperature, which suggests an increasing fraction of crystalline material and a reduced fraction of amorphous material with increasing temperature and/or different sizes of the crystallites.
- At a given temperature, a saturation of the increase in hardness can be observed over time. Hence, it can be assumed that processes changing the microstructure of Parylene C are time-limited and endure until a certain fraction of crystalline domains and/or a certain crystallite size is reached.
- Considering that the increase in the hardness observed for a non-accelerated aged sample and a non-aged sample is significantly different after thermal annealing, it can be assumed that the processes changing the microstructure of Parylene C are irreversible, and the final state in the material depends on the thermal history of the material after deposition; e.g., an immediate thermal annealing after deposition could cause a larger number of smaller crystallites leading to higher hardness, compared to a sample that was aged in a non-accelerated way and annealed later featuring a smaller number of larger crystallites and, hence, a lower hardness (assuming that saturation of crystallinity was reached each).

The validation or refutation of these assumptions as well as a more detailed investigation of the aging mechanism of Parylene C has to be the focus of future studies.

## 5. Conclusions

The presented study shows that Parylene C is a highly stable material without any impact of non-accelerated aging over ~1800 days on its barrier properties, chemical bonds, and optical properties. Different deposition conditions, Parylene C thicknesses, and various post-treatments, such as annealing, UV treatment, as well as sterilization by steam or electron beam, show only minor impact on the freshly prepared Parylene C and no impact on its aging process. Comparing the various post-treatments, however, an impact of the UV post-treatment on the initial Parylene C structure before aging is observed: The Parylene C is oxidized and the cutoff in the UV-VIS spectrum is shifted to higher wavelengths. This effect has to be considered or can be intentionally used when processing Parylene C with processes involving UV light. The impact of non-accelerated aging on the mechanical properties is comparably small, with an increase in the Young’s modulus by ~10% and no change in the hardness. However, for both mechanical quantities an influence of the temperature is observed. Hence, the usage of accelerated aging methods in order to investigate the reliability of Parylene C might cause misleading results and has to be carefully evaluated.

In summary, the experiments show that Parylene C is excellently suitable for long-term usage in applications, such as MEMS. Furthermore, it is compatible with the conditions of most microtechnologies, even though UV-based processes, such as lithography, can cause small material changes.

## Figures and Tables

**Table 1:** Overview of the thicknesses, deposition conditions, and post-treatments of the different Parylene C samples for the investigation of the barrier properties, optical properties, and chemical stability. The varied parameter is highlighted in bold.

Parylene Thickness [µm]	Deposition Rate/Sublimation Temperature	Post-Treatment Parameters
**2.15 ± 0.03**	Regular/130 °C	None
**4.23 ± 0.13**	Regular/130 °C	None
**5.84 ± 0.13**	Regular/130 °C	None
1.82 ± 0.01	**Slow/100 °C**	None
2.20 ± 0.16	Regular/130 °C	**Steam sterilization**
2.36 ± 0.01	Regular/130 °C	**Electron beam sterilization**
1.82 ± 0.04	Regular/130 °C	**Annealing at 200 °C**
2.19 ± 0.06	Regular/130 °C	**UV treatment**

**Table 2:** Overview of the samples used for investigation of the aging impact on the mechanical properties.

Sample	Parameters and Description
Initial	Measurement after deposition
5 years non-accelerated aging	Storage in clean room at 22 °C for ~1800 days
1 year accelerated aging	Storage in oven at 87 °C for 97 h in air
3 years accelerated aging	Storage in oven at 87 °C for 291 h in air
5 years accelerated aging	Storage in oven at 87 °C for 472 h in air
Sintered	Sintering at 115 °C for 100 h in air
5 years non-accelerated aging and sintered	Storage in clean room at 22 °C for ~1800 days and subsequent sintering at 115 °C for 100 h in air

**Table 3:** Overview of the samples used for investigation of the aging impact on the mechanical properties.

		Post-Treatment	
		dependent	independent
Non-accelerated aging	dependent	Young’s modulus	
	independent	Optical properties when UV post-treated Chemical composition when UV post-treated Hardness	Optical properties (except for UV post-treatment) Chemical composition (except for UV post-treatment) Barrier properties

**Figure A3:**
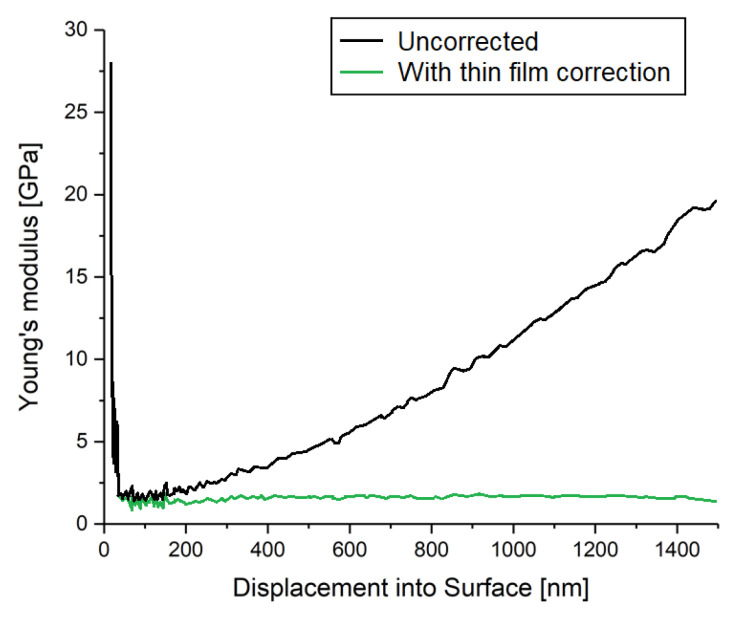
Impact of the thin film correction, i.e., considering and subtracting the silicon substrate.

## Data Availability

Not applicable.

## Appendix A

The accelerated aging can be converted into equivalent non-accelerated aging at ambient or body temperature using the Arrhenius reaction function given in Equation (A1) with the reaction rate k, the activation energy E_a_, the gas constant R, the temperature T, and the constant A [6].

(A1) $$k=A\;\cdot {\;e\;}^{-\frac{E_{a}}{\mathrm{RT}}}$$

For accelerated aging, the accelerating factor F can be obtained by dividing two reaction rates k_1_ and k_2_ calculated for two different temperatures T_1_ and T_2_ using Equation (A1). For polymers, a “10-degree rule” according to Equation (A2) is commonly used to approximate the Arrhenius relationship, assuming a first-order or pseudo-first order aging process [6,36,37].

(A2) $$F=\frac{k_{1}}{k_{2}}{=2\;}^{\frac{T_{1}-{\;T}_{2}}{10}}$$

The “10-degree rule” according to Equation (A2) states that for every temperature increase in 10 K the reaction and aging rate will double [6,36,37].

## Appendix B

**Table A1:** Comparison of Parylene thicknesses calculated by assuming constructive interference according to Equation (2) in the measured FTIR spectra and the Parylene thicknesses measured by profilometry and reflectometry, respectively. For the calculation, a refractive index of *n* = 1.56 was used.

Sample Description			Calculated Parylene Thickness				
Parylene Thickness by Profilometry/Reflectormetry [µm]	Deposition	Post- Treatment	Wave Number at Maximum [cm^−1^]	Wavelength [µm]	Integer i	Parylene Thickness [µm]	Average Parylene Thickness [µm]
2.15 ± 0.03	Regular	None	3732	2.68	2	1.72	1.77 ± 0.07
			1768	5.66	1	1.81	
4.23 ± 0.13	Regular	None	2490	4.02	3	3.86	3.91 ± 0.08
			1657	6.04	2	3.87	
			799	12.52	1	4.01	
5.84 ± 0.13	Regular	None	2027	4.93	4	6.32	6.33 ± 0.04
			1527	6.55	3	6.30	
			1005	9.95	2	6.38	
1.82 ± 0.01	Slow	None	3364	2.97	2	1.91	1.69 ± 0.31
			2177	4.59	1	1.47	
2.20 ± 0.16	Regular	Steam Sterilization	2966	3.37	2	2.16	2.15 ± 0.01
			1495	6.69	1	2.14	
2.36 ± 0.01	Regular	ElectronBeam Sterilization	3929	2.55	3	2.45	2.62 ± 0.18
			2449	4.08	2	2.62	
			1145	8.73	1	2.80	
1.82 ± 0.04	Regular	Annealing	2777	3.60	2	2.31	2.27 ± 0.05
			1430	6.99	1	2.24	
2.19 ± 0.06	Regular	UV treatment	2839	3.52	2	2.26	2.19 ± 0.09
			1508	6.63	1	2.13	

## Appendix C

Nano-indentation in general monitors the displacement of a tip into the sample surface in dependency on the load. From the results, a contact depth is calculated considering the geometry and stiffness of the contact. The latter is calculated from the derivation of the force and depth at maximum depth at the unloading segment. The CSM method in particular superimposes a periodic oscillation with a frequency of 45 Hz and an amplitude of 2 nm with the actual indentation process. This allows a more precise measurement of the stiffness due to multiple unloading curves across the indentation curve. Hence, a more reliable value for the indentation hardness can be achieved. As depicted in Figure A3, the thin film method takes the response of the silicon substrate into account to correct the calculated values for Young’s modulus and hardness.

## Appendix E

**Table A2:** Assignment of the FTIR vibrational modes.

Wave Number k [cm^−1^]	Mode	Reference
826	H−C−H two neighboring hydrogen atoms bonded to the ring	[56]
826	C−C aliphatic stretching	[57]
826	C−H out-of-plane wagging	[42,53]
878, 906, 949	C−H single hydrogen to the ring, out-of-plane wagging	[42,53,56]
1004, 1050	C−H in-plane bending	[42,53]
1050	C−Cl bonding to the ring	[56]
1077, 1110, 1158, 1210, 1267	CH_2_ wagging	[42,53]
1341, 1404, 1495	C=C semicircle stretching	[42,53]
1452	CH_2_ on the benzene ring	[56]
1452	CH_2_ deformation	[42,53]
1493	C−C aromatic stretching	[57]
1558, 1610	C=C ring stretching	[42,53]
1700	Parylene Oxidation (C=O stretching)	[32,42,53]
1745, 1897	Summation bands due to out-of-plane C−H wagging modes	[42,53]
2865, 2928	C−H aliphatic stretching	[42,53,56,57]
3020	C−H aliphatic stretching	[42,53]
3020	C−H aromatic stretching	[56,57]
